# Expression and Characteristics of Two Glucose-Tolerant GH1 β-glucosidases From *Actinomadura amylolytica* YIM 77502^T^ for Promoting Cellulose Degradation

**DOI:** 10.3389/fmicb.2018.03149

**Published:** 2018-12-18

**Authors:** Yi-Rui Yin, Peng Sang, Wen-Dong Xian, Xin Li, Jian-Yu Jiao, Lan Liu, Wael N. Hozzein, Min Xiao, Wen-Jun Li

**Affiliations:** ^1^State Key Laboratory of Biocontrol and Guangdong Provincial Key Laboratory of Plant Resources, School of Life Sciences, Sun Yat-sen University, Guangzhou, China; ^2^College of Agriculture and Biological Science, Dali University, Dali, China; ^3^Bioproducts Research Chair, Department of Zoology, College of Science, King Saud University, Riyadh, Saudi Arabia; ^4^Department of Botany and Microbiology, Faculty of Science, Beni-Suef University, Beni-Suef, Egypt; ^5^Key Laboratory of Biogeography and Bioresource in Arid Land, Xinjiang Institute of Ecology and Geography, Chinese Academy of Sciences, Ürümqi, China

**Keywords:** *Actinomadura amylolytica*, glucose tolerance, β-glucosidase, GH1, cellulose degradation

## Abstract

The bioconversion of lignocellulose in various industrial processes, such as biofuel production, requires the degradation of cellulose. *Actinomadura amylolytica* YIM 77502^T^ is an aerobic, Gram-positive actinomycete that can efficiently degrade crystalline cellulose by extracellular cellulases. Genomic analysis of *A. amylolytica* identified 9 cellulase and 11 β-glucosidase genes that could potentially encode proteins that digest cellulose. Extracellular proteome characterization of *A. amylolytica* cell-free culture supernatant by liquid chromatography tandem mass spectrometry analysis revealed that 4 of these cellulases and 2 of these β-glucosidases functioned during cellulose hydrolysis. Thin-layer chromatography analysis revealed extracellular β-glucosidases play a major role in carboxyl methyl cellulose (CMC) degradation of products in culture supernatants. In this study, 2 of the identified secreted β-glucosidases, AaBGL1 and AaBGL2, were functionally expressed in *Escherichia coli* and found to have β-glucosidase activity with wide substrate specificities, including for *p*-nitrophenyl β-D-glucopyranoside (pNPG), *p*-nitrophenyl-beta-D-cellobioside (pNPC), and cellobiose. Moreover, AaBGL1 and AaBGL2 had high tolerances for glucose. After adding these β-glucosidases to commercial cellulases, the degradation rates of CMC, Avicel, birch sawdust, and corncob powder increased by 37, 42, 33, and 9%, respectively. Overall, this work identifies an alternative potential source of β-glucosidases with potential applications in commercial cellulose utilization and the bioenergy industry.

## Introduction

Cellulose is an ecologically friendly material, the main component of lignocellulosic materials, especially agro-industrial residues, such as wheat straw, rice straw, corn stover, bagasse, and wood chips, and comprises 35–50% of a plant’s dry weight (Khandeparker and Numan, 2008; Brinchi et al., 2013). Cellulose is a polysaccharide consisting of a backbone of β-1,4-glucose units. A series of enzymes is essential for the complete degradation of cellulose into glucose, including endo-1,4-β-glucanases (endoglucanase, EC 3.2.1.4), exo-1,4-β-glucanases (i.e., cellobiohydrolases; EC 3.2.1.91), and β-glucosidases (EC 3.2.1.21). Endoglucanases and cellobiohydrolases act on internal cellulose chains and cellulose chain ends to release smaller fragments and cellobiose, respectively, and then the cello-oligosaccharides are ultimately degraded to glucose by β-glucosidases. The accumulation of cello-oligosaccharides, such as cellobiose and cellotriose, inhibits the functioning of both endoglucanases and cellobiohydrolases during simultaneous saccharification and, therefore, β-glucosidases play a crucial role in enzymatic degradation of cellulose by relieving product inhibition of cello-oligosaccharides for cellulases (Chauve et al., 2010).

A variety of microorganisms, including filamentous fungi, bacteria, and archaea, have the ability to produce cellulases (Jayant et al., 2011; Thomas et al., 2017). The filamentous fungus *Trichoderma reesei* (i.e., anamorph of *Hypocrea jecorina*) is a cellulase overproducer widely used in commercial and industrial lignocellulose degradation and bioethanol production (Henrissat et al., 1985; Huang et al., 2014). It secretes various endoglucanases, exoglucanases and beta-glucosidases to break down and convert cellulose into glucose (Ouyang et al., 2006; Peterson and Nevalainen, 2012). However, the β-glucosidase activity of these cellulase mixtures is too low to prevent inhibition by cellobiohydrolase following cellobiose accumulation (Saloheimo et al., 2002; Zhang et al., 2010). The low β-glucosidase activity of the extracellular secretions of *T. reesei* limits their application in degradation of cellulosic biomass (Nieves et al., 1998; Rahman et al., 2009). Therefore, the discovery of additional β-glucosidases is necessary to enhance the efficiency of cellulose hydrolysis.

Based on analysis of sequences, enzymatic properties, and three-dimensional protein structures, β-glucosidases that hydrolyze substrates through double displacement mechanisms, which also permit enzymes to transglycosylate, are classified as members of glycoside hydrolase (GH) families 1, 3, 5, 9, 16, 30, and 116 (Thongpoo et al., 2013)^1^. A large number of β-glucosidases from the GH1 and GH3 families have been purified from microorganisms and characterized (Wang et al., 2005). Most bacterial β-glucosidases employed in cellulose hydrolysis belong to the GH1 family (Cantarel et al., 2009). A majority of these bacterial β-glucosidases are sensitive to glucose, with only a few being glucose tolerant (Bohlin et al., 2010; Datta, 2016), which is a major barrier to efficient utilization of cellulose (Mallerman et al., 2014). To effectively hydrolyze cellulose and accumulate high levels of monosaccharides during enzymatic hydrolysis of lignocelluloses, the GH1 β-glucosidase should have a high glucose tolerance.

In the work presented here, two secreted GH1 β-glucosidases, AaBGL1 and AaBGL2, were identified by secretome and genomic analyses of *Actinomadura amylolytica* YIM 77502^T^. AaBGL1 and AaBGL2 were then functionally expressed in *Escherichia coli* BL21 and the resulting recombinant proteins were purified and characterized. Both AaBGL1 and AaBGL2 can degrade cellobiose and *p*-nitrophenyl β-D-glucopyranoside (pNPG). AaBGL1 can also effectively degrade *p*-nitrophenyl β-D-cellobioside (pNPC). Furthermore, AaBGL1 and AaBGL2 are highly tolerant of glucose. After adding these β-glucosidases to commercial cellulase, the degradation rate of carboxyl methyl cellulose (CMC), Avicel, corncob powder, and birch sawdust increased. The demand for β-glucosidases that are insensitive to glucose is increasing with commercial cellulase utilization. Compared with other β-glucosidases, both AaBGL1 and AaBGL2 had high Ki values. These results suggest GH1 β-glucosidases secreted from *A. amylolytica* have potential applications in industrial cellulose utilization.

## Materials and Methods

### Strains, Plasmid, and Culture Conditions

*Actinomadura amylolytica* YIM 77502^T^ (i.e., DSM 45822^T^ and CCTCC AA 2012024^T^) was stored in our laboratory (Jiao et al., 2015). The *E. coli* DH5α strain (Invitrogen, United States) was used for cloning and the *E. coli* BL21 DE3 strain (Invitrogen, United States) was utilized as the host for expression of proteins. The pET28a vector (Invitrogen, United States) was used to construct recombinant plasmids. *A. amylolytica* YIM 77502^T^ was cultured on R2A medium (0.5 g/l yeast extract, 0.5 g/l peptone, 0.5 g/l glucose, 0.5 g/l soluble starch, 0.5 g/l casein acid hydrolysates, 0.5 g/l sodium pyruvate, 0.3 g/l K2HPO4, and 0.024 g/l MgSO4) at 40°C. Recombinant *E. coli* strains were grown at 37°C in lysogeny broth (LB) medium (1% tryptone, 0.5% yeast extract, and 1% sodium chloride; pH 7.0) containing kanamycin (50 μg/ml).

### Genome Sequencing and Assembly and Gene Functions

*Actinomadura amylolytica* YIM 77502^T^ was cultured in R2A and then genomic DNA was extracted using a MasterPure Gram-positive DNA Purification kit (Epicentre MGP04100) following the standard DNA isolation procedure recommended by the manufacturer with modifications (Wu et al., 2009). Whole-genome sequencing was performed on the Illumina HiSeq 2500-PE125 platform with massively parallel sequencing Illumina technology at the Beijing Novogene Bioinformatics Technology Co., Ltd. All high-quality paired reads were assembled using the SOAP *de novo*^2^ into a number of scaffolds (Gu et al., 2013). Glimmer v3.0 (Chen et al., 2011) was used for gene prediction in assembled sequences of *A. amylolytica* YIM 77502^T^. The sequence data described here have been deposited in GenBank (Accession number: CP032402). Proteins encoded by *A. amylolytica* were annotated using cluster of orthologous groups (COG) protein functional classification (Tatusov et al., 2003). Carbohydrate-active enzymes (CAZymes) of *A. amylolytica* YIM 77502^T^ were identified using the CAZyme Analysis Toolkit^3^ (Petit et al., 2015). The glycoside hydrolase families were analyzed using HMMER software based on the Pfam database^4^ (Finn et al., 2011).

### Determination of Cellulose Degradation Activity of *A. amylolytica* Fermentation Broth

Degradation of filter paper was assessed by inoculating strain *A. amylolytica* YIM 77502^T^ into a test tube containing filter paper basic medium (NaH_2_PO_4_, KNO_3_, and MgSO_4_) after static culture at 40°C for 1 month. *A. amylolytica* was cultured in cellulose basic medium (NaH_2_PO_4_, KNO_3_, MgSO_4_, and Avicel 2 g/l) at 40°C with shaking at 180 rpm for 14 days. The supernatant was harvested by centrifuging for 10 min at 12,000 × *g* at 4°C.

The cellulose degradation activity of the above fermentation broth was measured using CMC (Sigma, United States), Avicel (Sigma, United States), and cellobiose (Sigma, United States). Fermentation broth (100 μl) was added to 400 μl PBS buffer (pH 7) containing 1% (w/v) CMC or Avicel to test cellulase activity. This mixture was incubated at different temperatures (25–90°C) for 1 h. The release of reducing sugar from CMC was determined using the 3,5-dinitrosalicylic acid assay and measuring the absorbance at 540 nm (Miller, 1959). One unit (IU) of CMCase activity was defined as the volume of fermentation broth required to release 1 μmol of reducing sugar from CMC per minute. Hydrolytic products of the fermentation broth from CMC were identified by thin-layer chromatography (TLC) using silica gel 60 plates (Merck, Darmstadt, Germany) developed with 1-butanol/acetic acid/water (2:1:1, v/v/v). Sugars were detected by heat treatment at 120°C for 10 min after spraying the plates with freshly prepared 5% (v/v) H_2_SO_4_ in ethanol (Yin et al., 2015). Glucose (G1), cellobiose (G2), cellotriose (G3), and cellotetrose (G4) were used as sugar standards. The sugar standards was purchased from Sigma, United States.

The β-glucosidase activity of the fermentation broth was measured using cellobiose. Fermentation broth (100 μl) was added to 400 μl PBS buffer (pH 7) containing 1% (w/v) cellobiose at different temperatures (25–80°C) for 30 min to test β-glucosidase activity. The release of glucose from cellobiose was determined based on the absorbance at 490 nm of the mixture using a Glucose Oxidase Assay Kit (Abnova, China). One unit (IU) of β-glucosidase activity was defined as the volume of fermentation broth required to release 2 μmol glucose from cellobiose per minute.

### LC-MS/MS Analysis of Secreted Glycoside Hydrolases

The above fermentation broth was transferred into 5 kD ultra-filter concentrators (Sartorius, Germany) and centrifuged for 30 min at 4°C in a swing bucket rotor, resulting in the medium become concentrated by 30-fold. Extracellular protein was precipitated overnight by ammonium sulfate precipitation. The precipitate was resuspended in 150 μL supernatant. Proteins were isolated and digested as described by Hornburg et al. (2014). Peptides were desalted on C18 StageTips as previously described and subjected to liquid chromatography tandem mass spectrometry (LC-MS/MS) analysis (Schwanhäusser et al., 2013). The peptides were then separated using a nano high-performance liquid chromatography (HPLC; Thermo Fisher Scientific) with a C18 column. HPLC was directly coupled to a quadrupole-Orbitrap mass spectrometer via a nano electrospray ion source (Q Exactive^TM^, Thermo Fisher Scientific). The mass spectrometry (MS) raw data was processed by MaxQuant (v. 1.3.8.2; Cox and Mann, 2008). MS/MS spectra were searched against the *A. amylolytica* YIM 77502^T^ genomic database.

### Gene Cloning and Plasmid Construction

The putative β-glucosidase genes *aabgl1* (1335 bp) and *aabgl2* (1431 bp) from the genomic sequences of *A. amylolytica* were amplified via polymerase chain reaction (PCR) with the four designed primers listed in Table 1 using TransStarFastPfu Fly DNA Polymerase (TransGen Biotech, China). The PCR cycle consisted of denaturation at 98°C for 3 min, followed by 34 cycles at 98°C for 10 s, 65°C for 20 s, and 72°C for 45 s, and then a final incubation at 72°C for 5 min for the final extension. The PCR products were cloned into the *pET*28a plasmid, which had been digested with *BamH* I and *Hind* III, using the *pEASY*-Uni Seamless Cloning and Assembly Kit (TransGen Biotech, China). Recombinant plasmids with the correct size fragments were sequenced. Subsequently, the DNA from the correct recombinant plasmids were transformed into *E. coli* BL21 (DE3) for protein expression.

**Table 1:** Primer sets used in this study.

Primer name	Sequence (5′→3′)	Primer length (bp)
Aabgl1-F	GACAGCAAATGGGT CGCGGAATGAACCTT CCCGCCGACT	39
Aabgl1-R	TGCTCGAGTGCGGCC GCATCATGGGGCT CCTCTGTGG	37
Aabgl2-F	GACAGCAAATGGGT CGCGGAATGACAG CACACGAGACGC	39
Aabgl2-R	TGCTCGAGTGCGGC CGCATCAGTCCGG CAGTCCGCC	36


*All primers were designed by Primer Premier 5. The underlined region represents the homologous recombinant fragment with the vector.*

### Sequence Analysis

DNA and protein sequences were aligned using the BLASTx and BLASTp programs (Madden, 2002)^5^, respectively. Signal peptides were predicted using SignalP (Bendtsen et al., 2004)^6^. The primary structures of the amino acid sequences were deduced and analyzed using EXPASY tools^7^. Multiple alignments with protein sequences of the closely related AaBGL1 and AaBGL2 (retrieved from NCBI database) were carried out using Clustal X (Thompson et al., 1997). Phylogenetic analyses were performed using the MEGA 5 software package (Tamura et al., 2011). Trees were constructed using the maximum likelihood (ML) method with a Poisson correction model. Structural models of AaBGL1 and AaBGL2 were generated with the MODELLER package (Sali and Blundell, 1993) using the β-glucosidase *BglM-G1 mutant H75R* (PDB ID, 5NS7; sequence identity, 47%) from a marine metagenome and β-xylosidase (PDB ID, 1GNX; sequence identity, 52%) from *Streptomyces* sp. as templates.

### Expression, Production, and Purification of Recombinant β-glucosidases

To express the recombinant AaBGL1 and AaBGL2 proteins, the above transformants were cultured overnight in LB medium containing 100 μg/ml kanamycin at 37°C and with shaking at 220 rpm. One ml of overnight culture was inoculated into 100 ml fresh LB medium containing 100 μg/ml kanamycin and incubated at 37°C with shaking at 220 rpm. To induce expression of the recombinant β-glucosidases, 0.1 ml of 100 mM IPTG was added to the cell suspension until the cells reached mid-exponential phase (OD_600_≈0.6) and incubated at 25°C with shaking at 220 rpm for 6 h. Cell pellets were harvested by centrifuging at 8,000 × *g* at 4°C for 10 min and resuspended in 20 ml buffer A (20 mM sodium phosphate, 300 mM NaCl, and 10 mM Tris; pH 8.0).

The resuspended cells were disrupted by ultrasonication. The lysates were centrifuged at 12000 × *g* for 30 min at 4°C. Cell-free extracts were transferred onto a Ni-chelating affinity column (GE, United States) as the recombinant proteins possessed an N-terminal His-tag. The column was then washed with ten column volumes of buffer A and then ten column volumes of buffer A containing 20 mM imidazole (pH 8.0) and eluted with buffer A containing 300 mM imidazole (pH 8.0). The eluted proteins were desalted using disposable PD-10 Desalting Columns (GE, United States) and the desalted proteins were used for enzyme characterization. Sodium dodecyl sulfate polyacrylamide gel electrophoresis (SDS-PAGE) was performed using a 10% polyacrylamide gel that was then stained with Coomassie brilliant blue dye R-250 (Liu et al., 2010). Protein concentrations were determined with Bradford Protein Assay Kit (Order NO. C503031, Sangon Biotech, China) using bovine serum albumin as the standard.

### Enzymatic Characterization of Recombinant β-glucosidases AaBGL1 and AaBGL2

#### Assays of Enzymatic Activity

β-glucosidase activity was assayed using cellobiose and *p*-nitrophenyl-β-D-glucopyranoside (pNPG). Activity against cellobiose was determined by adding 10 μg of purified protein to a 200-μl reaction mixture containing 2% (w/v) cellobiose. After 10 min, the activity was tested using a Glucose Oxidase Assay Kit (Abnova, China). One unit (U) of β-glucosidase activity was defined as the amount of enzyme required to release 2 μmol glucose from cellobiose per minute. When using pNPG as the substrate, 10 μg protein was added to a 200-μl reaction mixture containing 2.5 mM pNPG (Sigma, St. Louis, MO, United States). After 5 min of incubation at the optimal temperature, the reaction was stopped by adding 0.6 ml of 1 M Na_2_CO_3_ (Yang et al., 2015). The pNP was measured by monitoring the absorbance at 405 nm (Harnpicharnchai et al., 2009). One unit of β-glucosidase activity was equivalent to 1 μmol of pNP released from the pNPG in 1 min.

#### Optimal Temperatures and Thermostabilities

The optimal temperatures for AaBGL1 and AaBGL2 were determined by measuring β-glucosidases activity at different temperatures (10–80°C) at the optimal pH according to the activity assay method. For thermostability analysis, purified β-glucosidases were incubated in buffer A at temperatures ranging between 10 to 80°C for 30, 60, and 120 min. The samples were rapidly cooled in an ice-water bath and residual activity was measured by the standard method.

#### Optimal pHs and Stabilities at These pHs

The optimal pHs for purified recombinant AaBGL1 and AaBGL2 were investigated in pHs ranging from 2.0 to 11 in buffer (50 mM Na_2_HPO_4_-citric acid, pH 2.0–8.0; 50 mM Tris-HCl, pH 8.0–9.0; and 50 mM glycine-NaOH, pH 9.0–11.0). The stabilities of purified AaBGL1 and AaBGL2 at different pHs were assessed by incubating these enzymes in buffers with different pHs as described above at 4°C for 12 and 24 h. The amount of residual activity at the optimal temperature was then determined according to the activity assay method.

#### Effect of Metal Ions and Chemical Reagents on Enzymatic Activity

To evaluate the effects of metal ions and chemical reagents on enzymatic activity, 10 mM of various metal ions and chemical reagents (K^+^, Na^+^, Fe^3+^, Mg^2+^, Mn^2+^, Ca^2+^, Cu^2+^, Co^2+^, Zn^2+^, Ni^2+^, SDS, and EDTA) were added individually to the reaction system. The control was tested using the same process described above without any additive in the reaction mixture.

#### Effect of Glucose Concentration on Enzymatic Activity

To investigate the effect of the end product glucose on catalytic activity, the reaction was carried out in the presence of glucose concentrations ranging from 0 to 3000 mM. The concentration of the initial substrate pNPG was 1 mM. For the control, the same reaction system was used, but glucose was not added.

#### Determination of Substrate Specificity

To determine the substrate specificity of AaBGL1 and AaBGL2, cellobiose, beechwood xylan, CMC, Avicel, and different *p*-nitrophenyl derivatives, such as *p*-nitrophenyl-β-D-glucopyranoside (*p*NPG), *p*-nitrophenyl-α-D-glucopyranoside (*p*NP-α-G), *p*-nitrophenyl β-D-xylopyranoside (*p*NPX), and *p*-nitrophenyl-β-D-cellobioside (pNPC), were used as substrates to measure enzymatic activity. All these substrates were purchased from Sigma, United States. The release of pNP was determined by measuring the absorbance at 405 nm of the mixture using pNP as the standard. One unit (U) of activity was defined as the amount of enzyme released from 1 μmol pNP/min. β-glucosidase activity for cellobiose was tested using the Glucose Oxidase Assay Kit (Abnova, China). One unit (U) of β-glucosidase activity was defined as the amount of enzyme required to release 2 μmol glucose/min from cellobiose. Beechwood xylan, glucan, CMC, and Avicel were measured using the 3,5-dinitrosalicylic acid assay (Miller, 1959). One unit (U) of enzymatic activity was defined as the amount of enzyme required to release 1 μmol glucose or xylose-equivalent reducing sugars per minute. All the substrates were purchased from Sigma (St. Louis, MO, United States).

### Determination of Kinetic Constants

The kinetic constants (Vmax and Km) of AaBGL1 and AaBGL2 were determined using different concentrations of cellobiose (0.2–2 mg/ml) and pNPG (0.5–5.0 mg/ml) at the optimal pH and temperature for 5 min. The Vmax and Km of the β-glucosidases for cellobiose and pNPG were calculated using Lineweaver-Burk plots.

### Enzymatic Hydrolysis

Enzymatic hydrolysis of different lignocellulosic materials, such as CMC, Avicel, birch sawdust, and corncob, was performed by adding the appropriate cellulases (Sangon Biotech, China) and/or AaBGL1 and AaBGL2 to a 0.5 ml reaction system containing 50 mM Na_2_HPO_4_-citric acid buffer (pH 6.0) and 0.5 ml of 1% (w/v) substrate at 40 and 50°C for 24 h. The dosages of cellulases and β-glucosidases (AaBGL1 and AaBGL2) were 0.2 and 0.02 U/mg, respectively, based on the dry weight of substrate (Ye et al., 2017). Pretreatment of birch sawdust and corncob with ionic liquid (1-ethyl-3-methylimidazolium acetate) was carried out according to the method described in previous studies (Cheng et al., 2012). After enzymatic hydrolysis, the reactions were terminated by boiling for 10 min. The supernatants were collected by centrifugation at 4°C at 10000 × *g* for 10 min. The glucose contents of the hydrolysis liquors were measured using the Glucose Oxidase Assay Kit (Abnova, China).

## Results

### Features of the *A. amylolytica* YIM 77502^T^ Genome

The complete genome of *A. amylolytica* YIM 77502^T^ consisted of a single circular 6.8 Mbp chromosome, and a G+C content of 73.28%. The genome contained 6,373 coding sequences with an average length of 922 bp. The general genomic features of *A. amylolytica* are listed in Supplementary Table S1. Among the predicted genes, 95.8% (6,105 genes) were assigned a function and 4.2% (268 genes) had unknown functions. In addition, 86.4% of the sequence encoded genes, while 13.6% belonged to internal sequences. The functions of these genes were mainly gene transcription (590), signal transduction (373), amino acid transport and metabolism (354), carbohydrate transport and metabolism (344), lipid transport and metabolism (324), energy production and conversion (289), coenzyme transport and metabolism (284), and cell wall/membrane/envelope biogenesis (273), as shown in Supplementary Figure S1.

### Genes Encoding Carbohydrate-Active Enzymes

**FIGURE 1:**
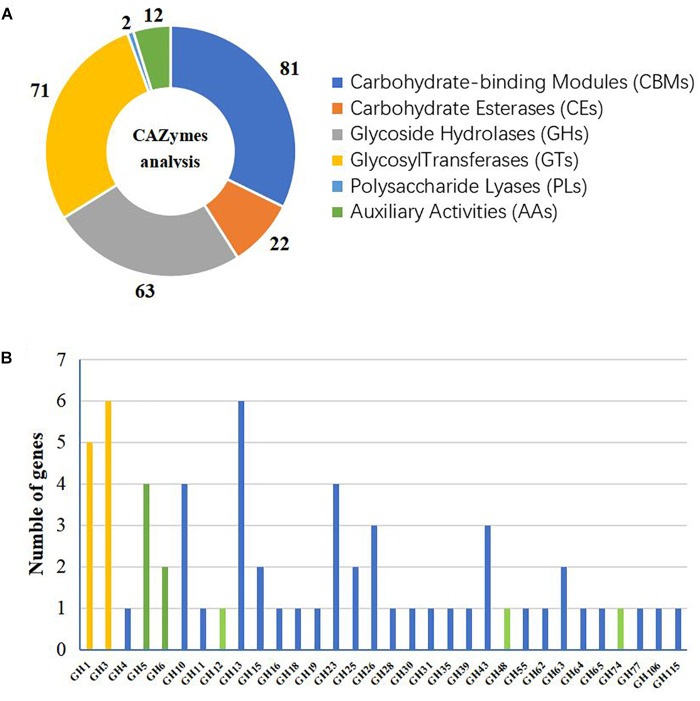
Glycoside hydrolase (GH) families of *Actinomadura amylolytica* YIM 77502^T^. **(A)** CAZyme analysis. **(B)** Number of GH genes. Histogram colors indicate different functions: yellow indicates predicted β-glucosidase, green indicates predicted cellulase, and blue indicates other predicted functions.

A CAZyme analysis was conducted to identify potential enzymes with plant cell-wall degradation ability. Through this analysis, 81 carbohydrate-binding modules (CBMs) distributed in 12 families, 63 glycoside hydrolases (GHs) in 32 families, 2 polysaccharide lyases (PLs) in 2 families, 22 carbohydrate esterases (CEs) in 5 families, 71 glycosyl transferases (GTs) in 14 families, and 12 auxiliary activities (AAs) in 4 families were found to be encoded in the genome of *A. amylolytica* (Figure 1A). GH1 (glycoside hydrolase family 1) and GH3 were predicted to function as β-glucosidases and GH5, GH6, GH12, GH48 and GH74 were predicted cellulases. Altogether, 11 β-glucosidase genes and 9 cellulase genes were predicted in the genome of *A. amylolytica* (Figure 1B).

### Cellulase Activity of *A. amylolytica* YIM 77502^T^

**FIGURE 2:**
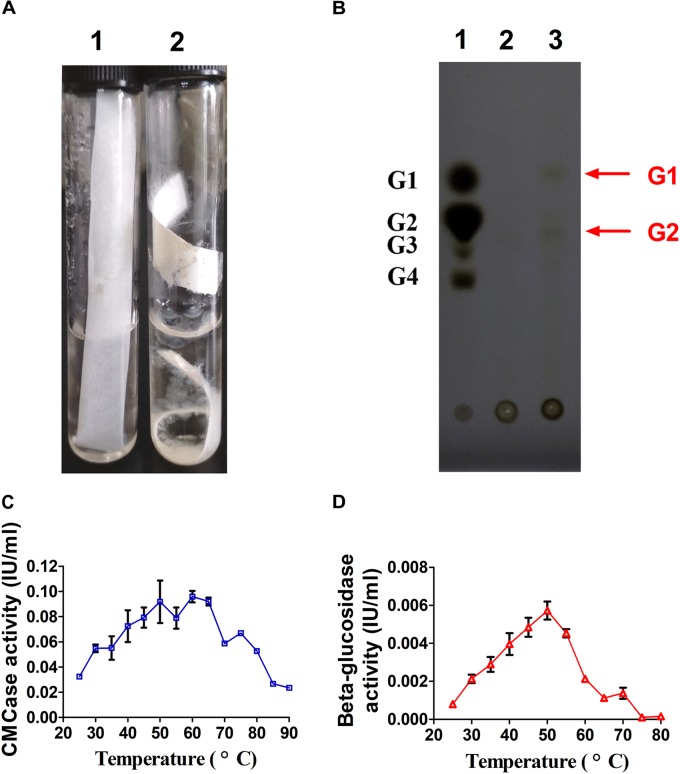
Cellulase activity of *A. amylolytica* YIM strain 77502^T^. **(A)** Hydrolysis experiment using filter paper. **(B)** Thin-layer chromatography plate analysis of hydrolytic products of carboxyl methyl cellulose (CMC) in fermentation broth of *A. amylolytica*, which was cultured with microcrystalline cellulose. **(C)** CMCase activity of fermentation broth. **(D)** β-glucosidase activity of fermentation broth.

As shown in Figure 2A, filter paper was degraded by *A. amylolytica* YIM 77502^T^ after culturing 1 month. TLC demonstrated the hydrolytic products of fermentation broth from CMC were mainly glucose and cellobiose (Figure 2B). Fermentation broths from cultures in microcrystalline cellulose basic medium for 2 weeks exhibited CMC and cellobiose activity under test conditions. High CMCase activity was found from 50 to 65°C, where the highest β-glucosidase activity was observed at 50°C (Figures 2C,D).

### Glycoside Hydrolases in the Secretome

**FIGURE 3:**
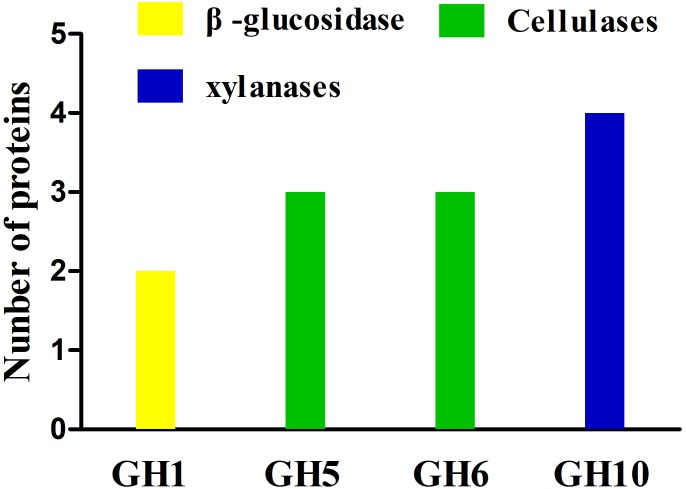
Glycoside hydrolases in the secretome of *A. amylolytica*.

The secretome of *A. amylolytica* was examined by MS. Secretome analysis revealed 209 proteins produced by *A. amylolytica*, which were mainly involved in carbohydrate transport and metabolism (43), amino acid transport and metabolism (23), protein turnover (18), cell wall/membrane/envelope biogenesis (16), energy production and conversion (10), and signal transduction (10), as shown in Supplementary Figure S2. Among these secreted proteins, 4 cellulases belonging to GH5 and GH6, 4 xylanases belonging to GH10, and two β-glucosidases belonging to GH1 were identified among the secretion proteins (Figure 3). These two β-glucosidases were designated AaBGL1 and AaBGL2.

### Cloning and Sequence Analysis of Genes *aabgl1* and *aabgl2*

**FIGURE 4:**
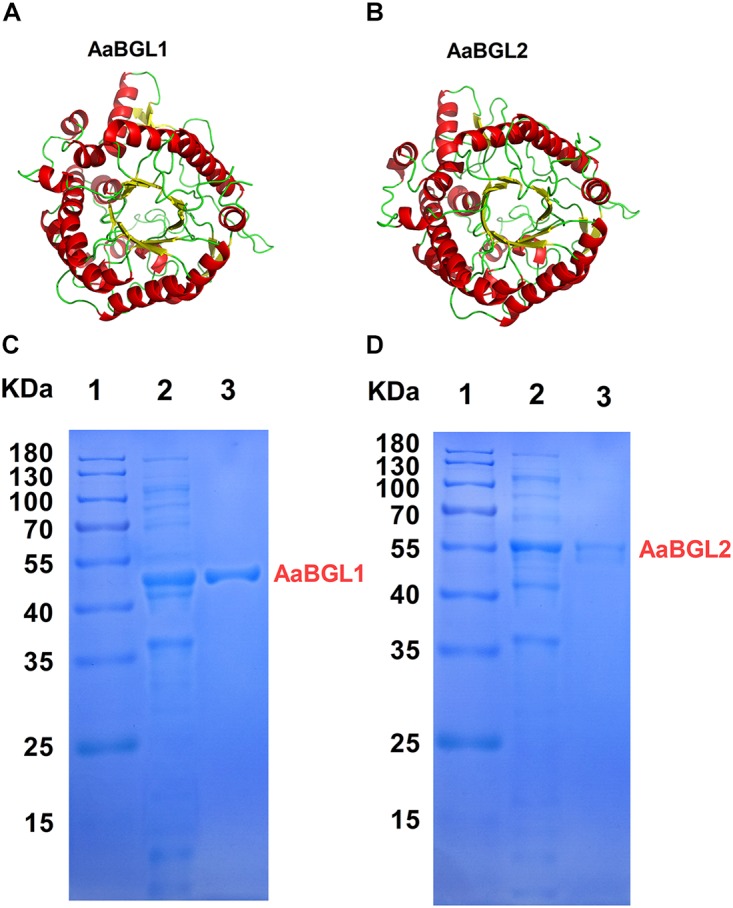
Three-dimensional model and purification of β-glucosidases AaBGL1 and AaBGL2. Structural models of **(A)** AaBGL1 and **(B)** AaBGL2. Sodium dodecyl sulfate polyacrylamide gel electrophoresis analysis of recombinant **(C)** AaBGL1 and **(D)** AaBGL2 produced by *E. coli* BL21. Lane 1, protein molecular weight marker, mass indicated on the left; lane 2, total protein in IPTG-induced *E. coli* BL21/*pET*28a-aabgl1 or *pET*28a-aabgl2; lane 3, purified AaBGL1 or AaBGL2.

According to the genome sequences of *A. amylolytica* YIM 77502^T^, two putative genes encoding β-glucosidases AaBGL1A (GenBank: MH974516) and AaBGL2 (GenBank: MH974517) were amplified by PCR and introduced into the *pET*28a vector to construct recombinant plasmids *pET28a-aabgl1* and *pET28a-aabgl2.* AaBGL1 and AaBGL2 consisted of 444 and 476 residues with theoretical molecular weights of 48.47 and 52.61 KDa, respectively. Signal peptides were not predicted at the N-termini of AaBGL1 and AaBGL2 based on analysis with Signal P 4.1 Server^8^. Sequence analysis revealed AaBGL1 and AaBGL2 contained distinct catalytic modules of glycosyl hydrolase family 1 (GH1) in the predicted enzyme proteins. The deduced amino acid sequence of AaBGL1 had the highest amino acid sequence identity (89%) to β-glucosidase (NCBI: WP 103938629.1) from *Actinomadura echinospora*. AaBGL2 had 84% similarity with β-glucosidases from *Thermomonospora curvata* (NCBI: WP 012852091.1) and *Actinomadura echinospora* (NCBI: WP 103937196.1). As shown in Figures 4A,B, the predicted three-dimensional model structures of AaBGL1 and AaBGL2 were very similar to other GH1 β-glucosidases with known structures The monomeric subunit of GH1 β-glucosidase adopts the expected topology of a single (α/β)_8_ barrel, with additional units of quite well-conserved secondary structures inserted between the α/β units. A phylogenetic analysis of the protein sequences revealed AaBGL1 and AaBGL2 also clustered with β-glucosidases (Supplementary Figures S3, S4).

### Expression and Purification of β-glucosidases AaBGL1 and AaBGL2

The β-glucosidase genes were successfully expressed in *E. coli* BL21 (DE3) and the resulting recombinant proteins with His-tagged N-termini were purified by Ni-NTA affinity chromatography. SDS-PAGE analysis indicated the molecular masses of the recombinant β-glucosidase proteins were in good agreement with the theoretical ones (Figures 4C,D).

### Enzymatic Characteristics of Purified Recombinant AaBGL1 and AaBGL2

#### Optimal Temperatures and Thermostabilities

**FIGURE 5:**
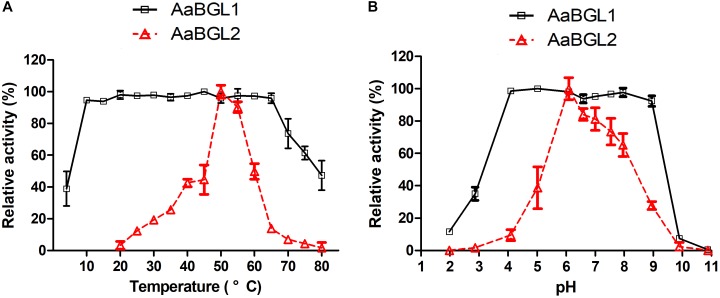
Effects of temperature and pH on the activity and stability of the recombinant AaBGL1 and AaBGL2. **(A,B)** Effect of **(A)** temperature and **(B)** pH on the activity of the recombinant β-glucosidases. The primary activity was designated 100%. Each value in the figure represents the mean ± standard deviation (*n* = 3). AaBGL1: 100% = 6.2 U/mg. AaBGL2 100% = 5.6 U/mg.

AaBGL1 exhibited high activity at 10–65°C at the optimal pH of 6.0. AaBGL2 exhibited the highest activity at 50°C and a pH of 6.0 (Figure 5A). Within 2 h, AaBGL1 and AaBGL2 were stable below 50°C, but their activity rapidly decreased when the temperature rose above 50°C (Supplementary Figures S5A, S6A). The thermostabilities of AaBGL1 and AaBGL2 were similar to reported β-glucosidases from fungi and bacteria, which were lost most activity when the temperature above 50°C (Santos et al., 2016; Florindo et al., 2018).

#### Optimal pHs and Stability at These pHs

The effects of pH on the activity of the β-glucosidases AaBGL1 and AaBGL2 were assessed. AaBGL1 functioned in a broad optimum pH, retaining more than 95% of the catalytic activity at pHs ranging from 4.0 to 9.0. The optimum pH of AaBGL2 was 6 (Figure 5B). As shown in Supplementary Figures S5B, S6B, AaBGL1 was stable in the pH range of 4.0–9.0, retaining 100% residual activity after incubating for 12 h in pH buffer at 25°C. AaBGL2 was relatively pH stable, retaining more than 60% residual activity after incubation for 24 h at pH 4–10 at 25°C.

#### Effects of Metal Ions and Chemical Reagents on Activity

The influence of various metal ions and chemical reagents on the activity of AaBGL1 and AaBGL2 was also investigated and the results are shown in Supplementary Figure S7. The enzymatic activity of AaBGL1 was 102 ± 0.3% and 100 ± 1.2% by K^+^ and Mg^2+^, respectively. Na^+^, Ca^2+^, Co^2+^, Ni^2+^, and SDS ions at a concentration of 10 mM did not affect AaBGL1 activity. Fe^3+^ and Mn^2+^ slightly inhibited both AaBGL1 and AaBGL2 activity, while Cu^2+^, Co^2+^, Zn^2+^, Ni^2+^, and EDTA severely inhibited AaBGL1 activity. The enzymatic activity of AaBGL2 was not influenced by K^+^, Na^+^, Mg^2+^, or Ca^2+^; however, the of addition Cu^2+^, Co^2+^, Zn^2+^, Ni^2+^, SDS, and EDTA strongly inhibited AaBGL2 activity.

#### Effect of Glucose Concentration on Enzymatic Activity

**FIGURE 6:**
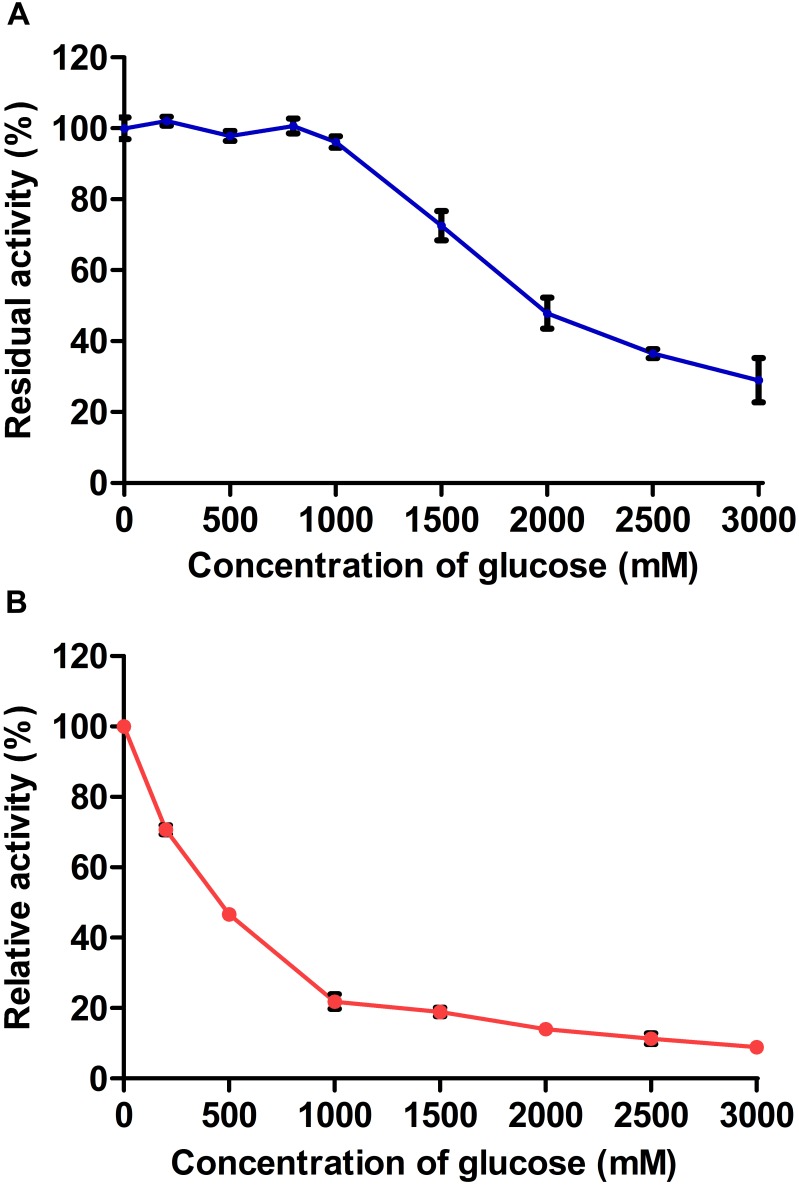
Effect of glucose concentration on recombinant **(A)** AaBGL1 and **(B)** AaBGL2.

The glucose tolerances of AaBGL1 and AaBGL2 were determined using 1 mM pNPG as a substrate. The activity of AaBGL1 and AaBGL2 measured in the absence of exogenous glucose was set as 100%. As shown in Figure 6, glucose had no effect on AaBGL1 activity when the added glucose concentration was less than 1000 mM. Even when the added glucose concentration was 2000 times greater than the pNPG concentration, AaBGL1 still retained more than 40% activity, suggesting AaBGL1 is highly tolerant of glucose. AaBGL2 also retained 40% activity when the added glucose concentration was 500 times greater than the pNPG concentration (Figure 6). This suggests AaBGL2 is also a glucose-tolerant β-glucosidase.

#### Substrate Specificities and Kinetic Constants of AaBGL1 and AaBGL2

Analysis of substrate specificity revealed AaBGL1 hydrolyzed pNPG, cellobiose, *p*NP-α-G, and pNPC, but not pNPX, beechwood xylan, CMC, or Avicel (Table 2). AaBGL2 exhibited activity for pNPG and cellobiose, but not other tested substrates. Based on Lineweaver-Burk plots (Supplementary Figures S8, S9), the Kcat, Km, and Vmax of AaBGL1 and AaBGL2 calculated using the pNPG and cellobiose as substrates are shown in Table 3.

**Table 2:** Substrate specificities of AaBGL1 and AaBGL2.

Substrates	AaBGL1 (U/mg)	AaBGL2 (U/mg)
Cellobiose	6.2 ± 0.3	5.6 ± 0.2
pNPG	4 ± 0.2	1.3 ± 0.1
*p*NP-α-G	0.56 ± 0.05	0
pNPX	0	0
pNPC	0.75 ± 0.1	0.1 ± 0.02
CMC	0	0
Avicel	0	0
Beechwood xylan	0	0


**Table 3:** Kinetic parameters of AaBGL1 and AaBGL2.

	Vmax (μmol/min/mg)		Km (μmol/ml)		Kcat (s^-1^)	
			
	Cellobiose	pNPG	Cellobiose	pNPG	Cellobiose	pNPG
AaBGL1	13.2	6.8	95.3	3.3	10.7	5.5
AaBGL2	18.9	1.9	187.7	0.73	16.6	1.7


### Potential Use of AaBGL1 and AaBGL2 for Enzymatic Hydrolysis of CMC, Avicel, and Ionic Liquid-Pretreated Birch Sawdust and Corncob Powder

**FIGURE 7:**
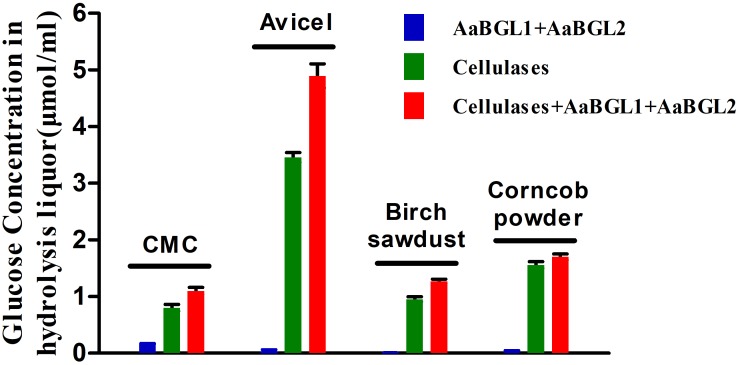
Cooperation of AaBGL1 and AaBGL2 with commercial cellulases in cellulosic material degradation.

To evaluate the potential use of AaBGL1 and AaBGL2 in degradation of lignocellulose, enzymatic hydrolysis of various lignocellulosic materials, including CMC, Avicel, and ionic liquid-pretreated birch sawdust and corncob powder, were performed. As shown in Figure 7, the glucose concentrations in the hydrolysis liquors were ordered Avicel > corncob > birch sawdust > CMC. After adding the β-glucosidases AaBGL1 and AaBGL2 to commercial cellulases, the degradation rates of CMC, Avicel, birch sawdust, and corncob powder increased by 37, 42, 33, and 9%, respectively. This suggests AaBGL1 and AaBGL2 cooperated with commercial cellulases in cellulose degradation.

## Discussion

### *A. amylolytica* Is a Potential Source of Cellulose Hydrolases

An aerobic Gram-positive actinomycete, *A. amylolytica* YIM 77502^T^ can digest cellulose rapidly and efficiently. A large number of glycosidase enzymes, including 9 cellulase genes and 11 β-glucosidase genes, have been predicted in the genome of *A. amylolytica*. Other actinomycetes, such as *Thermobifida fusca* (Wilson, 2010), *Thermobifida halotolerans* YIM 90462^T^ (Yin et al., 2015), *Thermoactinospora rubra* YIM 77501^T^ (Yin et al., 2017), and *Streptomyces* strains (Ventorino et al., 2016), also display highly lignocellulose-degrading activities. To date, many cellulose-degrading enzymes have been identified from cellulolytic actinomycetes (Vanee et al., 2017), indicating cellulolytic actinomycetes, such as *A. amylolytica*, are good potential sources of cellulose hydrolases.

In this research, 4 cellulases and 2 β-glucosidases were identified from the secretome of *A. amylolytica* YIM 77502^T^ cultured with microcrystalline cellulose. These enzymes may be involved in the degradation of cellulose, but no signal peptides were found in the 2 identified β-glucosidases, AaBGL1 and AaBGL2. Broeker et al. (2018) also found 32 proteins lacking signal peptides in the secretome of *Clostridium stercorarium*. These 2 β-glucosidases of *A. amylolytica* found in the secretome may be secreted through the release of proteins from cells through cell lysis or alternative secretion pathways. The sequence similarity, tertiary structures, and ML phylogenetic trees of AaBGL1 and AaBGL2 suggest GH1 β-glucosidases are conserved. Discovery of AaBGL1 and AaBGL2 in the secretome of *A. amylolytica* indicates LC-MS/MS is potentially a highly useful method of discovering new proteins with specific functions.

During cellulose degradation, cellulolytic microorganisms secrete some hydrolytic enzymes, including free cellulases (endoglucanase, cellobiohydrolases, and β-glucosidases) and cellulosome, which consist of a wide variety of polysaccharide-degrading enzymes (e.g., cellulases, hemicellulases, and pectinases) (Wilson, 2009; Artzi et al., 2017). Most bacteria and fungi hydrolyze cellulose by secreting free cellulases (Cavedon et al., 1990; Ja’afaru, 2013). Meanwhile, a cellulosome is a supramolecular multienzyme complex that can efficiently degrade lignocellulose and is found in just a few bacterial species, including *Clostridium* (*Ruminiclostridium*) *clariflavum* and *Clostridium* (*Ruminiclostridium*) *thermocellum* (Bayer et al., 2004; Shiratori et al., 2009; Shinoda et al., 2018). As described above, *A. amylolytica* YIM 77502^T^ secreted cellulases extracellularly to hydrolyze cellulose. A total of 344 and 38 genes in the genome of *A. amylolytica* were found to potentially be involved in carbohydrate transport and secretory transport of proteins, respectively (Supplementary Figure S1). Of these genes, membrane transporters are membrane proteins associated with transportation of macromolecules, such as proteins, across biological membranes (Saier et al., 2006). ATP-binding cassette transporters (ABC transporters) are members of a transport system superfamily and involved in the translocation of various substrates, such as glucose, cellobiose, and galactose, across membranes (Kemner et al., 1997; Petit et al., 2015).

**FIGURE 8:**
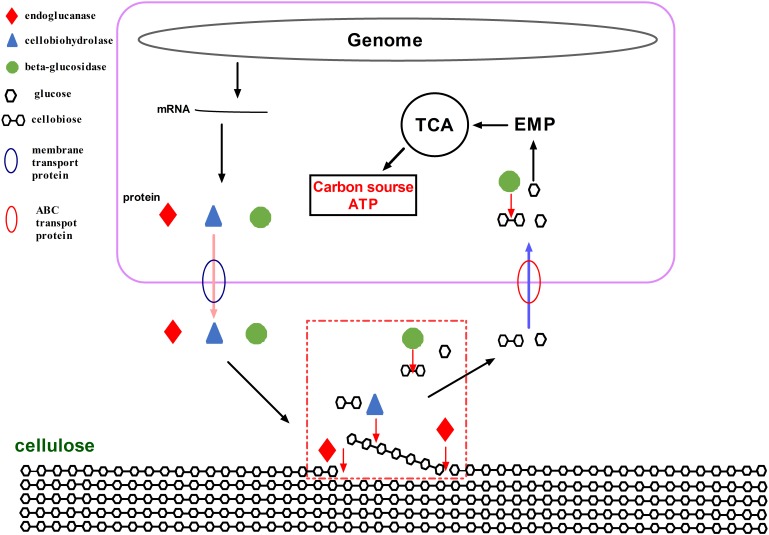
Schematic diagram of hydrolytic cellulose from *A. amylolytica* YIM 77502^T^.

Membrane transporters and ABC transporters from *A. amylolytica* possibly transfer cellulose-degrading enzymes and carbohydrates. Through the activity of *A. amylolytica* extracellular enzymes, secretome and TLC plate analyses of CMC hydrolysis products, including endoglucanase, cellobiohydrolases, and β-glucosidases, revealed hydrolysis of cellulose to oligosaccharides (cellobiose as the main product) and monosaccharide (glucose). As shown in a schematic diagram (Figure 8), cellulose-degrading enzymes are secreted into the extracellular medium and the cellulosic substrate is degraded by synergistic hydrolysis of these free glycosidolytic enzymes. The cellulose hydrolyze these products (cellobiose and glucose), which are then transported to the cytoplasm across the cell membrane through ABC transporters. The oligosaccharides are eventually hydrolyzed to monosaccharides by intracellular β-glucosidases. Finally, glucose entrance into the Embden-Meyerhof-Parnas pathway and tricarboxylic acid cycle provided material (carbon source) and energy (ATP) required for the growth and reproduction of *A. amylolytica*. This is the process of cellulose hydrolysis in pure culture in the laboratory simulation, but cellulose does not exist in a pure state in nature. Besides cellulose, there are also large amounts of hemicellulose, lignin, and pectin in plant biomass. Xylan, one of the main components of hemicellulose, can be hydrolyzed by xylanases. As shown in Figures 3, 4 GH10 xylanases were identified in the secretome of *A. amylolytica*, indicating they were also induced by cellulose. Robison reported that *Trichoderma reesei* Rut C-30 produces extracellular xylanases when grown on cellulose (Robison, 1984). The complete hydrolysis of lignocellulose requires the synergistic cooperation of cellulases, xylanase, pectinase, laccase, and other enzymes (Van Dyk and Pletschke, 2012). Therefore, for *A. amylolytica*, the collaborative expression of cellulases and xylanases may be a functional adaptation for hydrolysing plant biomass to be used as a carbon source.

Two β-glucosidases (AaBGL1 and AaBGL2) secreted by *A. amylolytica* exhibited synergistic cooperation with commercial cellulases. After adding AaBGL1 and AaBGL2, the decomposition rate of Avicel (40%), CMC (37%), and ionic liquid-pretreated birch sawdust (33%) increased by more than 30%. Meanwhile, the degradation rate of ionic liquid-pretreated corncob improved only 9%. Compared to wood, such as birch sawdust, corncob contains more hemicellulose as it is more than one-third of the dry matter of the corncob (Sun et al., 2014). This indicates degradation of substrate by the synergistic action of different kinds of cellulases is related to the purity or amount of cellulose in the substrate. It was also suggested that cellulases need to cooperate with other enzymes, such as hemicellulases (e.g., xylanase) and pectinase, when lignocellulose is degraded. The importance of the cocktail method for degradation of lignocellulosic materials also indicates that more new enzyme resources need to be found to enhance this synergistic degradation (Ibrahim, 2011).

The enzymatic properties of AaBGL1 and AaBGL2 include that, like other β-glucosidases, their activities were completely inhibited by Cu^2+^ (Chen et al., 2010; Wu et al., 2018). However, the activity of AaBGL1 was not affected by 10 mM SDS. A strong denaturant of proteins, SDS can inactivate most enzymes (Joo and Chang, 2010). The SDS stability of AaBGL1 suggests it is suitable for application in industrial purposes. During cellulose hydrolysis by cellulases, the hydrolysis products inhibit the activity of the enzymes. Most β-glucosidases are sensitive to the final product of glucose, which limits the use of β-glucosidase and efficient degradation of cellulose. The inhibition constants (Ki) of AaBGL1 and AaBGL2 were 1502 and 193.5 mM glucose, respectively. Meanwhile, the Ki of most fungal β-glucosidases, such as β-glucosidases from *Penicillium citrinum* UFV1, *Chaetomium globosum*, and *Neurospora crassa*, are between 0.1 and 10 mM (Bohlin et al., 2010; Da Costa et al., 2016). This indicates AaBGL1 and AaBGL2 are glucose-tolerant β-glucosidases. The glucose-tolerances of β-glucosidases are of great significance due to increases in glucose concentration and the conversion rates of soluble fermentable sugars from cellulose degradation.

## Conclusion

*Actinomadura amylolytica* YIM 77502^T^ exhibited CMCase and β-glucosidase activity when cultivated at 40°C using Avicel as the sole carbon source. Two GH1 β-glucosidases, designated AaBGL1 and AaBGL2, were identified in the secretome of *A. amylolytica* by LC-MS/MS. AaBGL1 and AaBGL2 were successfully expressed in *E. coli* BL21, and the recombinant proteins were purified and characterized. Both AaBGL1 and AaBGL2 were highly glucose-tolerant β-glucosidases and exhibited synergistic cooperation with commercial cellulases. Overall, the β-glucosidases studied in this work (AaBGL1 and AaBGL2) had different specificities and characteristics and could be used in different biotechnological applications, such as bioethanol production.

## Author Contributions

Y-RY, MX, and W-JL conceived the study. PS and WH were responsible for bioinformatics analysis of the genome and secretome. W-DX and XL cultured strains and collected samples. Y-RY and J-YJ separated proteins. LL and MX measured enzymatic activity. Y-RY and PS performed the data analysis and mapping. Y-RY, PS, MX, and W-JL wrote the manuscript. All authors discussed the results and commented on the manuscript. All authors read and approved the final manuscript.

## Conflict of Interest Statement

The authors declare that the research was conducted in the absence of any commercial or financial relationships that could be construed as a potential conflict of interest.

## Footnotes

1 http://www.cazy.org/

2 http://soap.genomics.org.cn/soapdenovo.html

3 http://allie.dbcls.jp/cooccur/CAT;CAZymes+Analysis+Toolkit.html

4 http://pfam.xfam.org/

5 http://blast.ncbi.nlm.nih.gov/Blast.cgi

6 http://www.cbs.dtu.dk/services/SignalP/

7 http://web.expasy.org/protparam/

8 http://www.cbs.dtu.dk/services/SignalP/

## Supplementary Material

FIGURE S1 Clusters of orthologous groups (COG) functional classification of *Actinomadura amylolytica* genome-encoded proteins.

FIGURE S2 COG functional classification of secretome of *A. amylolytica*.

FIGURE S3 Phylogenetic dendrogram obtained by maximum likelihood (ML) analysis based on amino acid sequences showing the phylogenetic position of AaBGL1 with related β-glucosidases. Bootstrap values (expressed as a percentage of 1000 replications) are given at nodes.

FIGURE S4 Phylogenetic dendrogram obtained by maximum likelihood (ML) analysis based on amino acid sequences showing the phylogenetic position of AaBGL2 with related β-glucosidases. Bootstrap values (expressed as a percentage of 1000 replications) are given at nodes.

FIGURE S5 Effects of temperature and pH on the stability of recombinant AaBGL1. **(A,B)** The effect of **(A)** temperature and **(B)** pH on stability.

FIGURE S6 Effects of temperature and pH on the stability of recombinant AaBGL2. **(A,B)** The effect of **(A)** temperature and **(B)** pH on stability.

FIGURE S7 Effects of metal ions and reagents on the activity of **(A)** AaBGL1 and **(B)** AaBGL2. Statistical analysis was performed using one-way ANOVA followed by Tukey’s test for comparison of multiple treatment groups. Data marked with ^∗^ and ^∗∗^ were statistically significant different at the *p* < 0.05 and *p* < 0.001 cut-off values, respectively.

FIGURE S8 Lineweaver-Burk plots of AaBGL1 for pPNG **(A)** and cellobiose **(B)**.

FIGURE S9 Lineweaver-Burk plots of AaBGL2 for pPNG **(A)** and cellobiose **(B)**.

TABLE S1 The general genomic features of *A. amylolytica.*
