# Supplementary figures and images for: Expression and Characteristics of Two Glucose-Tolerant GH1 β-glucosidases From Actinomadura amylolytica YIM 77502T for Promoting Cellulose Degradation
Source: Front Microbiol. 2018 Dec 18;9:3149. doi: 10.3389/fmicb.2018.03149 (PMC6305311; doi:10.3389/fmicb.2018.03149)

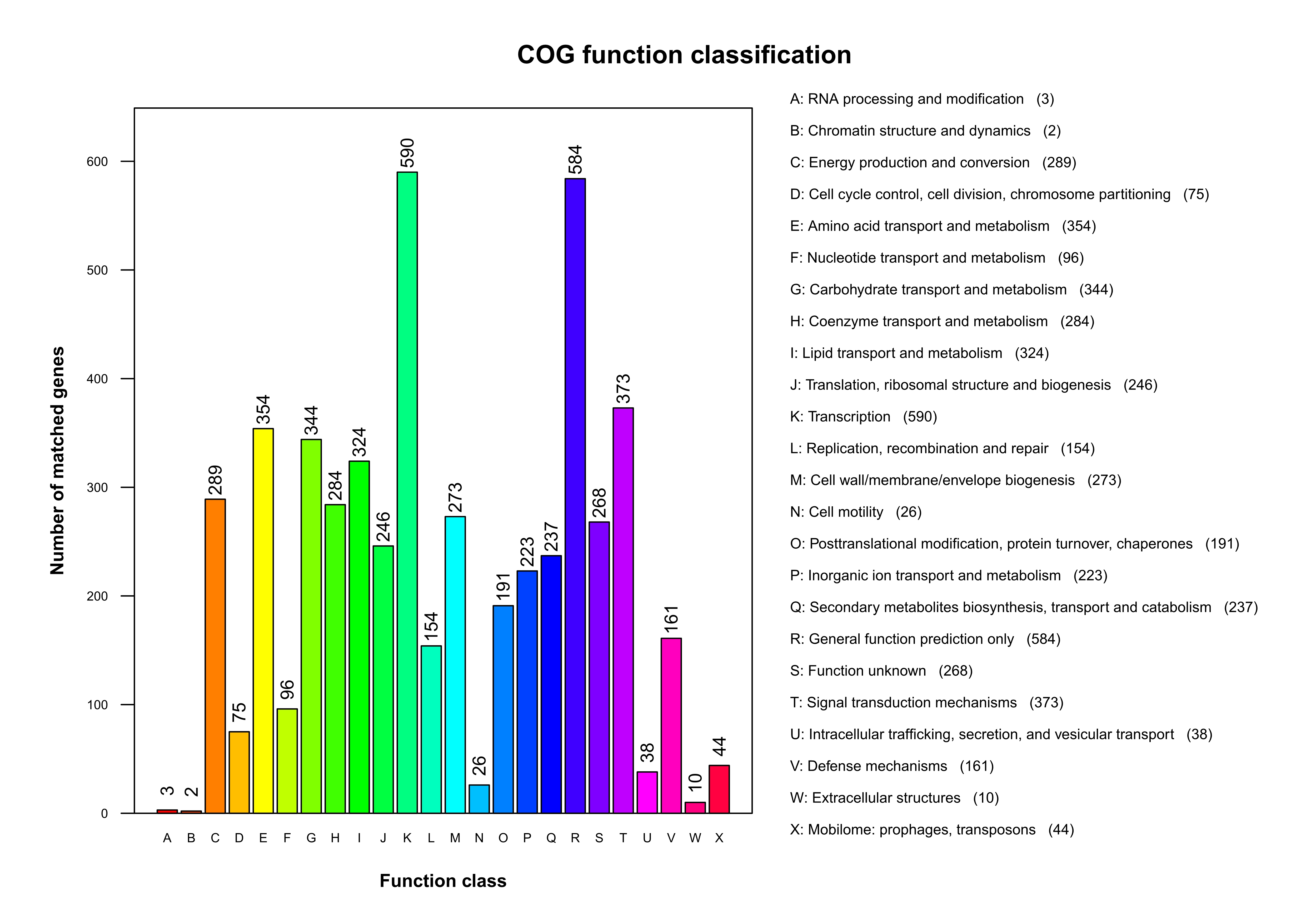

Supplement: FIGURE S1 — Clusters of orthologous groups (COG) functional classification of Actinomadura amylolytica genome-encoded proteins. [file Image_1.JPEG]

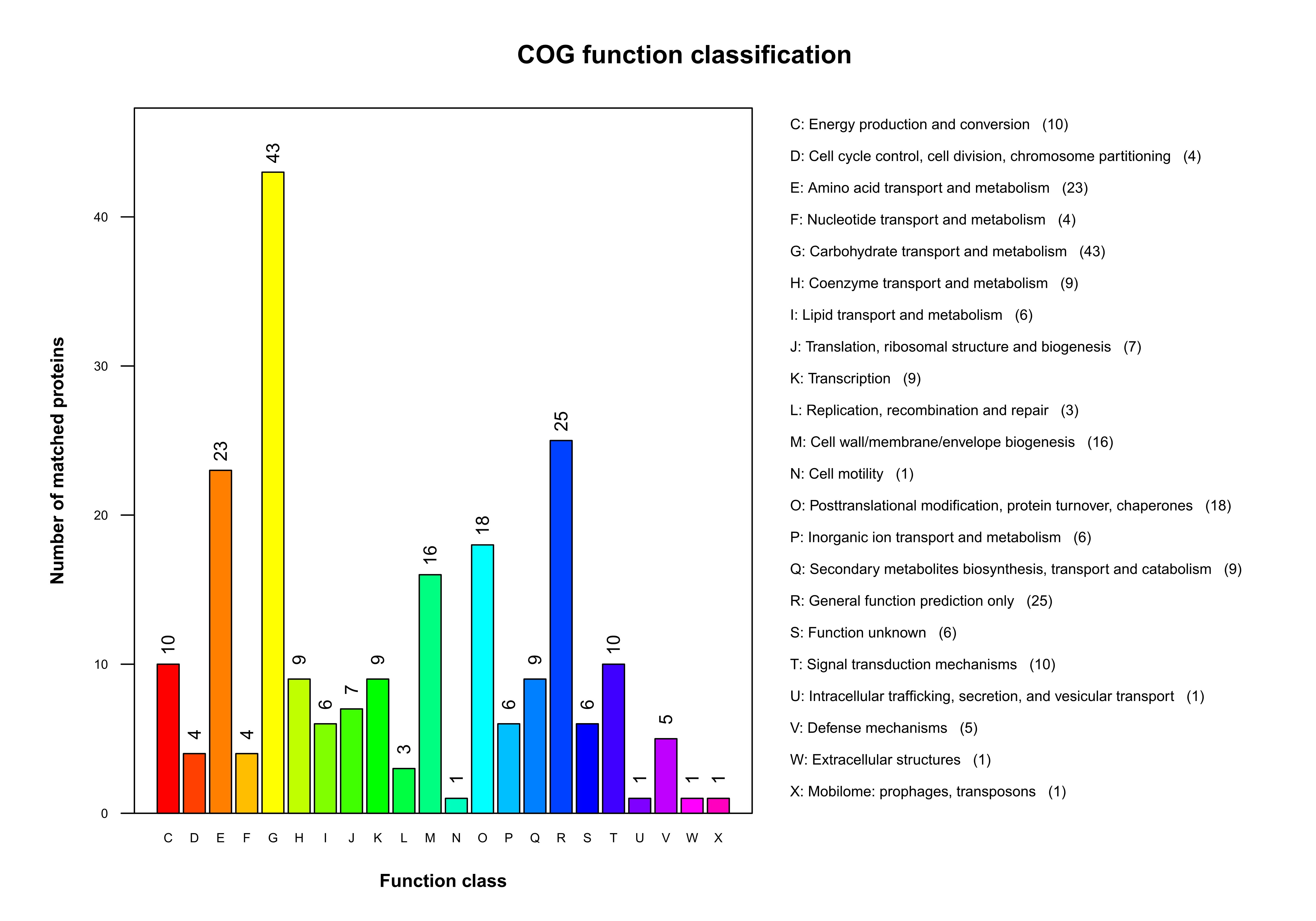

Supplement: FIGURE S2 — COG functional classification of secretome of A. amylolytica. [file Image_2.JPEG]

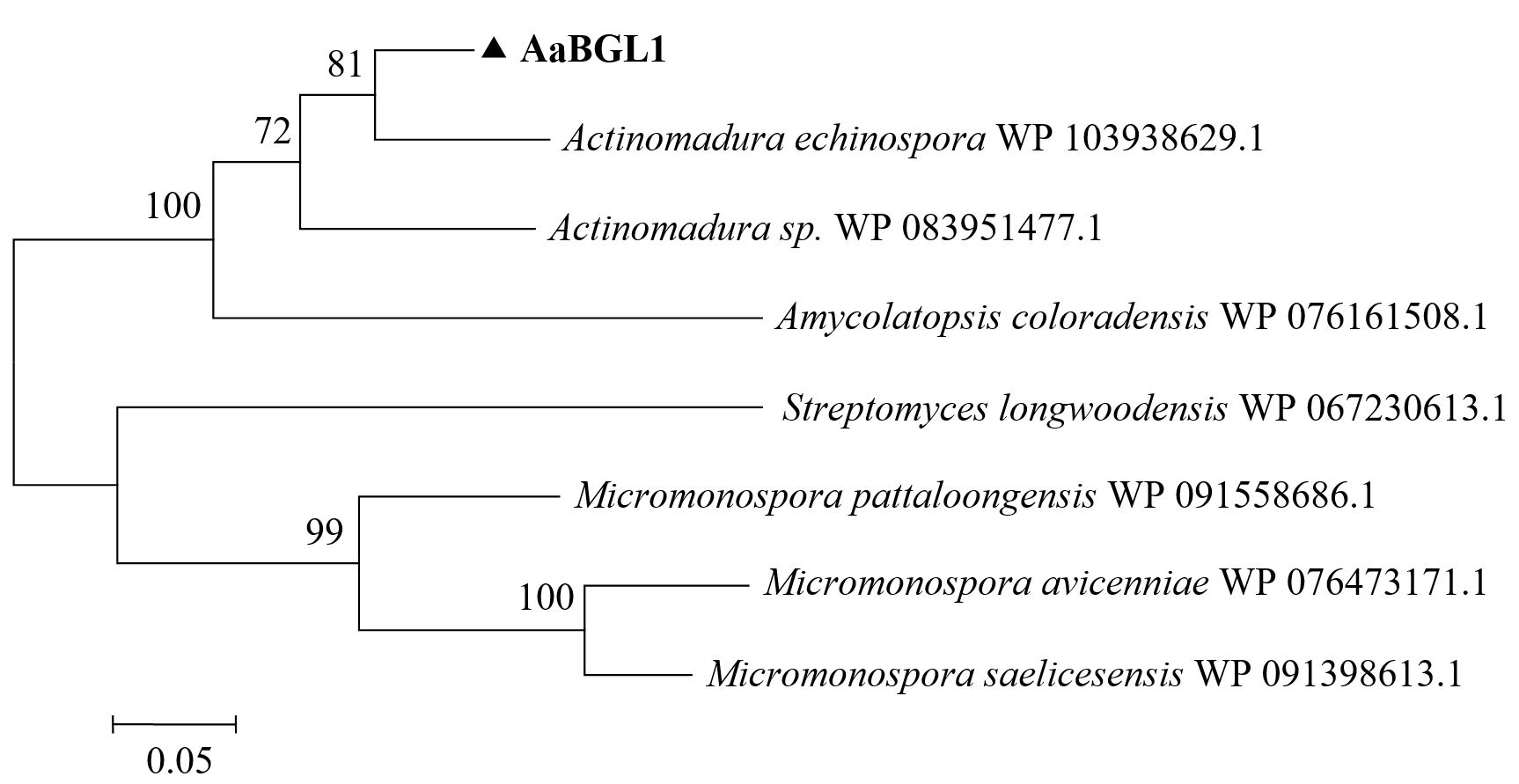

Supplement: FIGURE S3 — Phylogenetic dendrogram obtained by maximum likelihood (ML) analysis based on amino acid sequences showing the phylogenetic position of AaBGL1 with related β-glucosidases. Bootstrap values (expressed as a percentage of 1000 replications) are given at nodes. [file Image_3.JPEG]

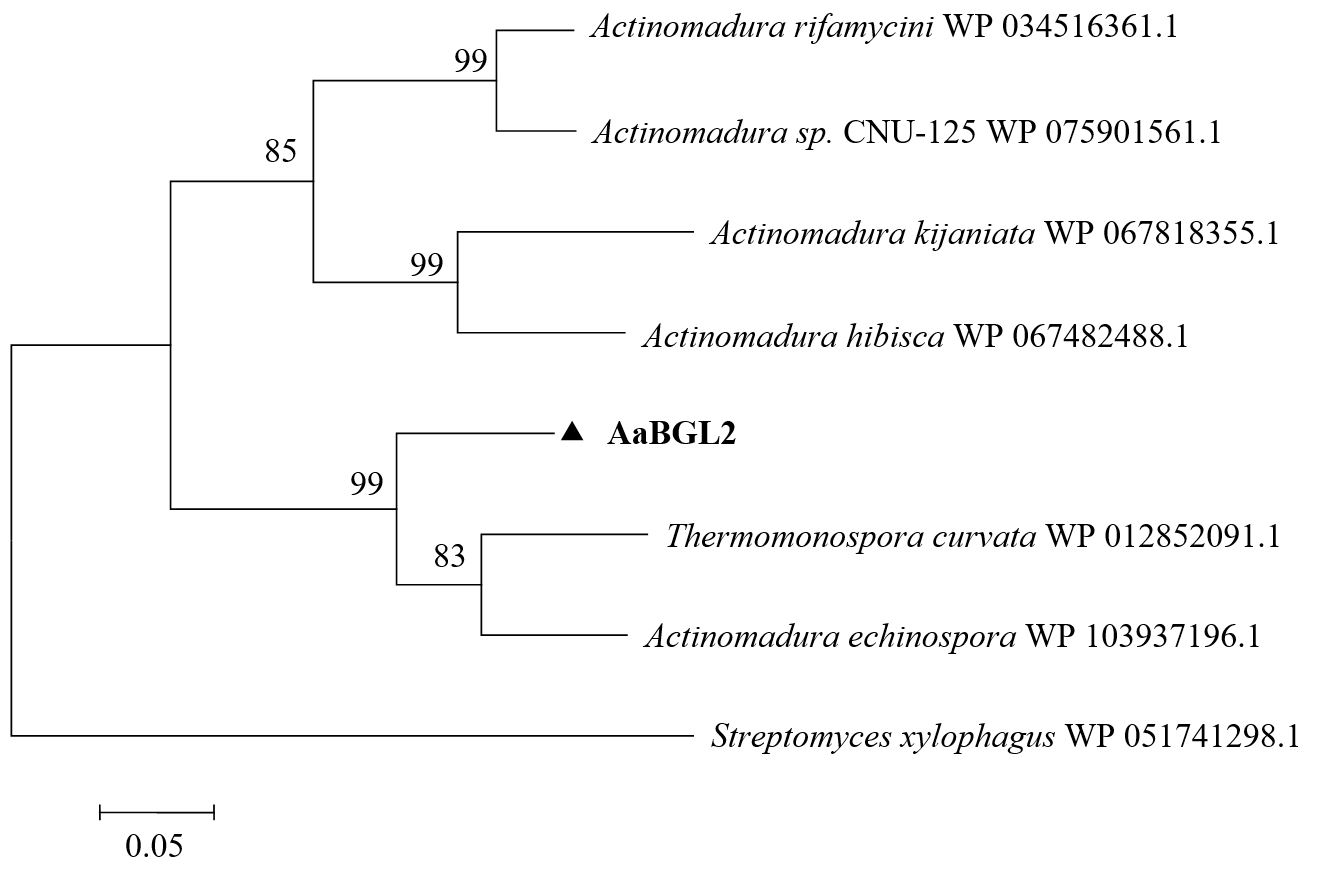

Supplement: FIGURE S4 — Phylogenetic dendrogram obtained by maximum likelihood (ML) analysis based on amino acid sequences showing the phylogenetic position of AaBGL2 with related β-glucosidases. Bootstrap values (expressed as a percentage of 1000 replications) are given at nodes. [file Image_4.JPEG]

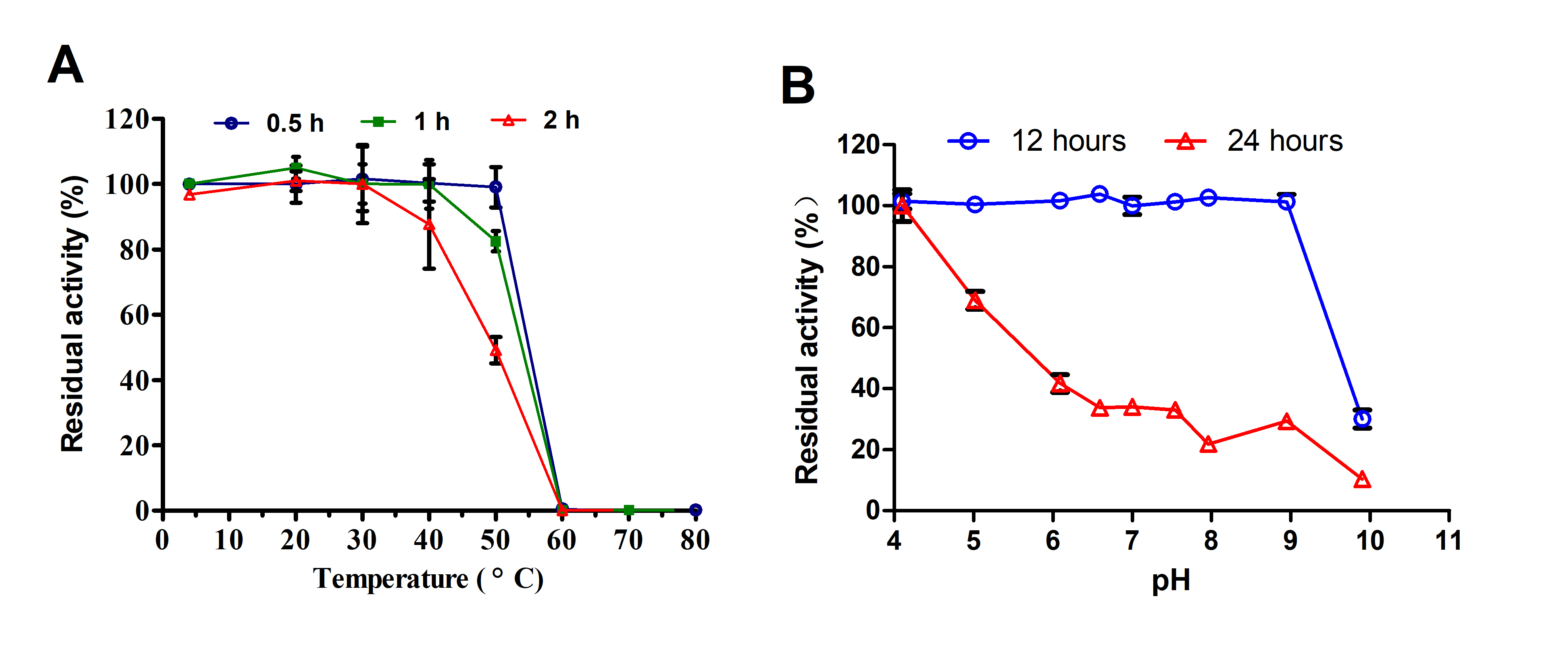

Supplement: FIGURE S5 — Effects of temperature and pH on the stability of recombinant AaBGL1. (A,B) The effect of (A) temperature and (B) pH on stability. [file Image_5.JPEG]

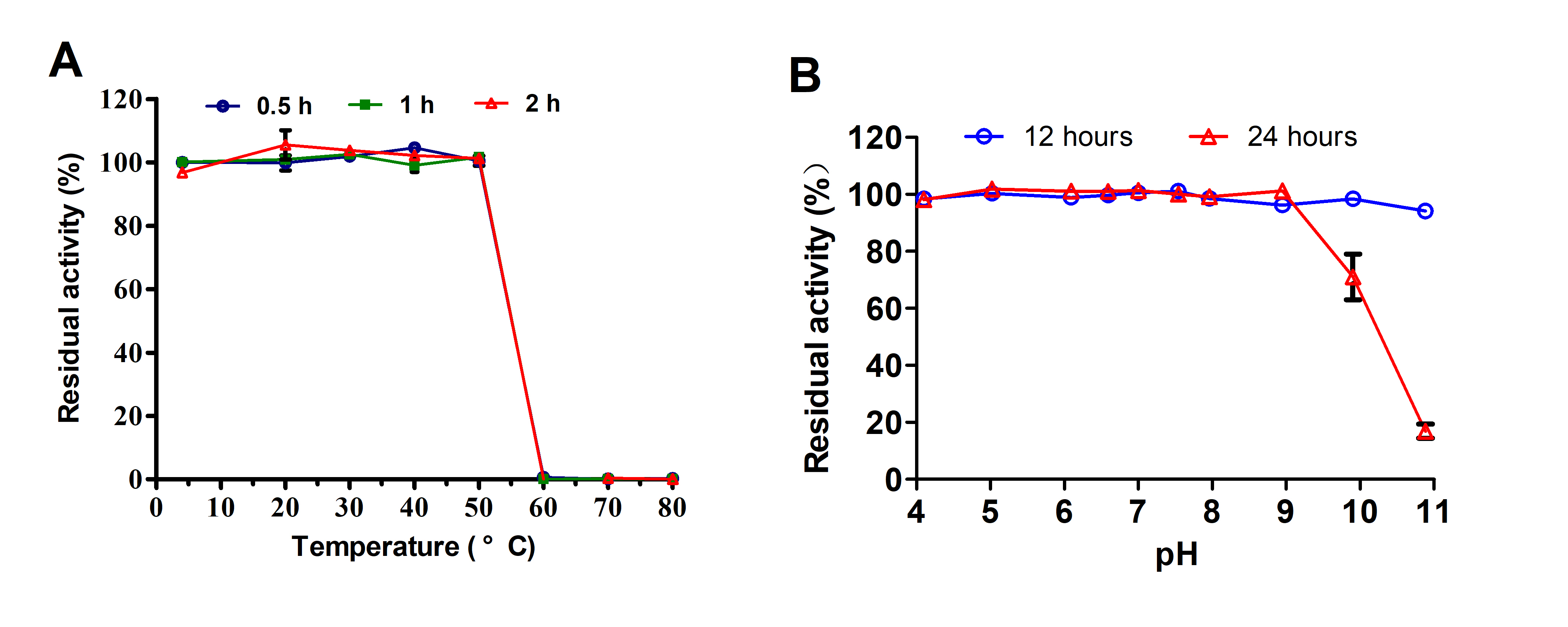

Supplement: FIGURE S6 — Effects of temperature and pH on the stability of recombinant AaBGL2. (A,B) The effect of (A) temperature and (B) pH on stability. [file Image_6.JPEG]

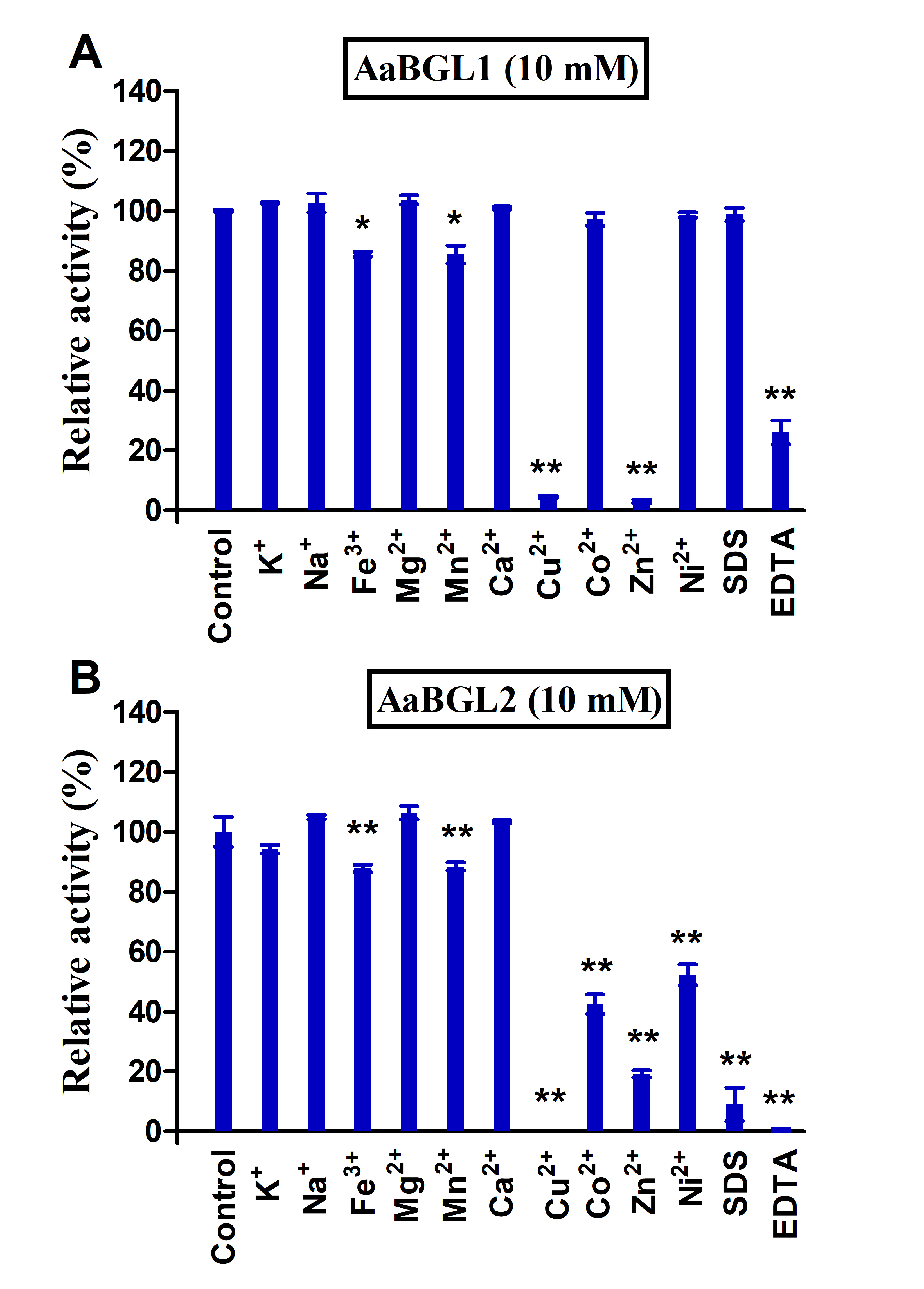

Supplement: FIGURE S7 — Effects of metal ions and reagents on the activity of (A) AaBGL1 and (B) AaBGL2. Statistical analysis was performed using one-way ANOVA followed by Tukey’s test for comparison of multiple treatment groups. Data marked with ∗ and ∗∗ were statistically significant different at the p < 0.05 and p < 0.001 cut-off values, respectively. [file Image_7.JPEG]

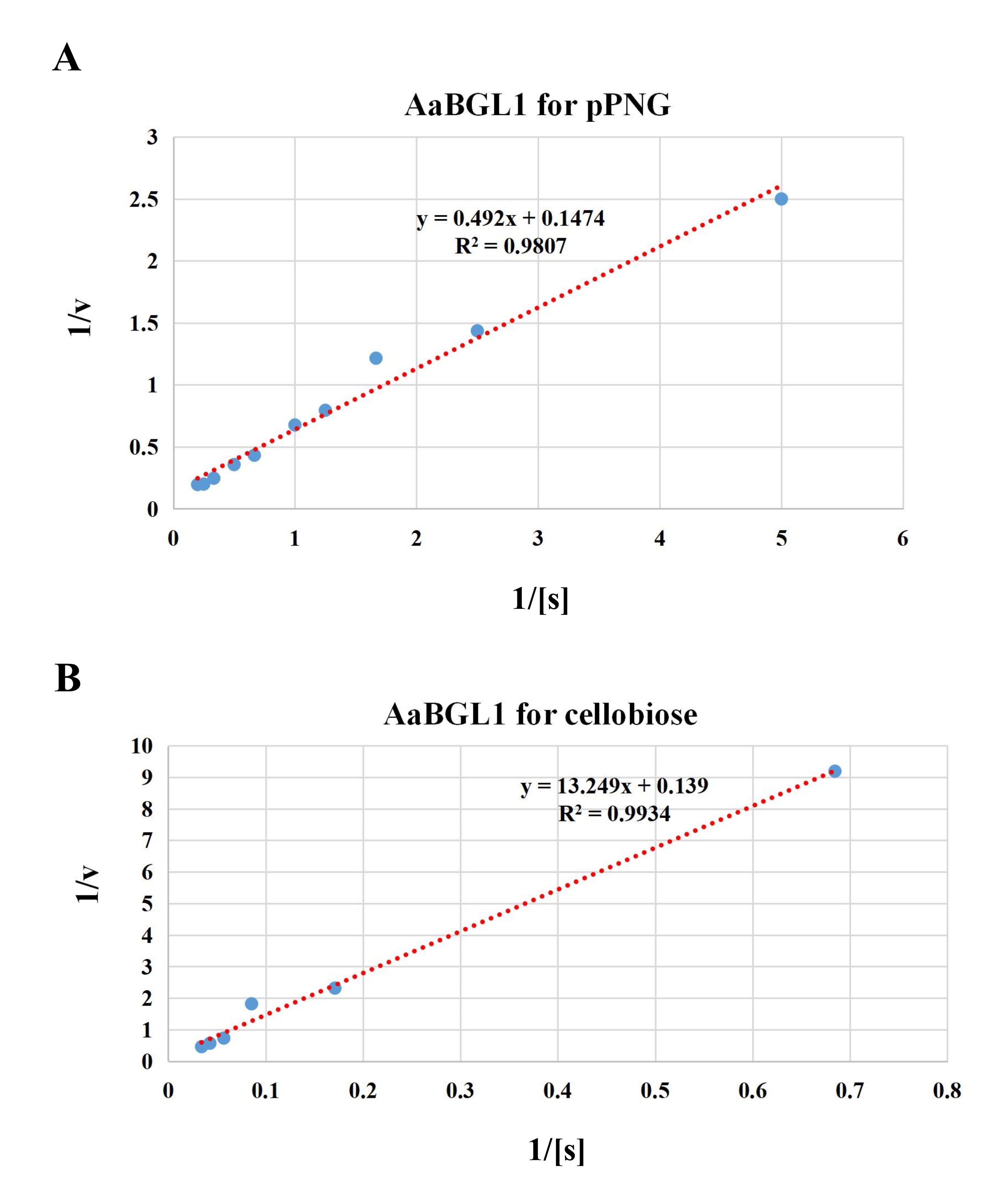

Supplement: FIGURE S8 — Lineweaver-Burk plots of AaBGL1 for pPNG (A) and cellobiose (B). [file Image_8.JPEG]

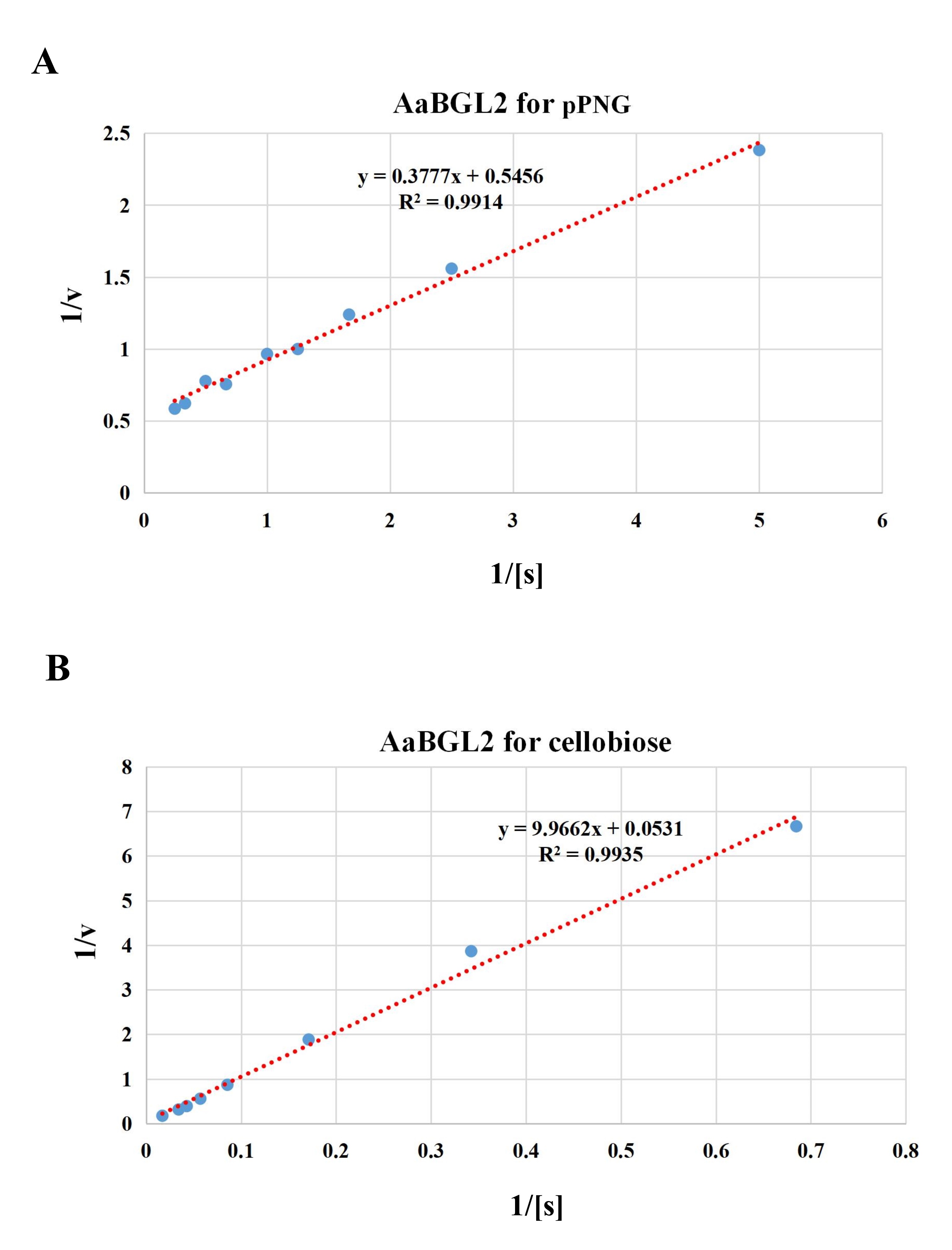

Supplement: FIGURE S9 — Lineweaver-Burk plots of AaBGL2 for pPNG (A) and cellobiose (B). [file Image_9.JPEG]
