# Supplementary material for: Expression and Characteristics of Two Glucose-Tolerant GH1 β-glucosidases From Actinomadura amylolytica YIM 77502T for Promoting Cellulose Degradation
Source: Front Microbiol. 2018 Dec 18;9:3149. doi: 10.3389/fmicb.2018.03149 (PMC6305311; doi:10.3389/fmicb.2018.03149)
Supplement: TABLE S1 — The general genomic features of A. amylolytica. [file Table_1.DOC]

**Supplementary material**

***Supplementary Table S1*** *The general genomic features of A. amylolytica.*

| Parameter | Values |
| --- | --- |
| Genome Size: | 6,800,142 bp |
| Gene Number: | 6373 |
| G+C Content: | 73.28% |
| Gene Average Length: | 922 bp |
| Gene Length: | 5,875,377 bp |
| % of Genome(Genes): | 86.4 |
| Gene Internal Length: | 924,765 |
| Gene Internal GC Content: | 69.82% |
| % of Genome(internal): | 13.6 |
| Known Function | 95.8% ( 6,105 genes) |
| UnKnown Function | 4.2% (268 genes) |
